# Microstructure and domain engineering of lithium niobate crystal films for integrated photonic applications

**DOI:** 10.1038/s41377-020-00434-0

**Published:** 2020-12-10

**Authors:** Dehui Sun, Yunwu Zhang, Dongzhou Wang, Wei Song, Xiaoyan Liu, Jinbo Pang, Deqiang Geng, Yuanhua Sang, Hong Liu

**Affiliations:** 1 Collaborative Innovation Center of Technology and Equipment for Biological Diagnosis and Therapy in Universities of Shandong, Institute for Advanced Interdisciplinary Research (iAIR), University of Jinan, Jinan, 250022 China; 2 Jinan Institute of Quantum Technology, Jinan, 250101 China; 3 CETC Deqing Huaying Electronics Co., Ltd., Huzhou, 313200 China; 4 Crystrong Photoelectric Technology Co., Ltd., Jinan, 250100 China; 5 State Key Laboratory of Crystal Materials, Shandong University, Jinan, 250100 China

**Keywords:** Optics and photonics, Microresonators

## Abstract

Recently, integrated photonics has attracted considerable interest owing to its wide application in optical communication and quantum technologies. Among the numerous photonic materials, lithium niobate film on insulator (LNOI) has become a promising photonic platform owing to its electro-optic and nonlinear optical properties along with ultralow-loss and high-confinement nanophotonic lithium niobate waveguides fabricated by the complementary metal–oxide–semiconductor (CMOS)-compatible microstructure engineering of LNOI. Furthermore, ferroelectric domain engineering in combination with nanophotonic waveguides on LNOI is gradually accelerating the development of integrated nonlinear photonics, which will play an important role in quantum technologies because of its ability to be integrated with the generation, processing, and auxiliary detection of the quantum states of light. Herein, we review the recent progress in CMOS-compatible microstructure engineering and domain engineering of LNOI for integrated lithium niobate photonics involving photonic modulation and nonlinear photonics. We believe that the great progress in integrated photonics on LNOI will lead to a new generation of techniques. Thus, there remains an urgent need for efficient methods for the preparation of LNOI that are suitable for large-scale and low-cost manufacturing of integrated photonic devices and systems.

## Photonics: Enabling new applications in optical communication and quantum technologies

A review of recent progress in the microstructure and domain engineering of lithium niobate film on insulator (LNOI) has concluded that it is a promising photonic material for developing integrated nonlinear photonic devices. The review, conducted by a team of researchers from China and led by Hong Liu from Shandong University, found that the high-performance electro-optic and nonlinear optical properties of LNOI makes it an ideal platform for integrated photonics. Furthermore, they also discovered that the microstructures could be constructed on LNOI platforms for photonic circuits using current manufacturing techniques such as complementary metal–oxide–semiconductor technology. The researchers concluded that the large-scale and low-cost manufacturing of integrated photonic devices and systems by mature manufacturing processes could lead to the development of new applications in optical communication and quantum technologies.

## Introduction

In contemporary society, the demand for high-bandwidth optical communication, including for mobile high-definition video streaming, autonomous vehicle applications, remote surgery, telepresence applications, and interactive 3D virtual reality gaming, is sharp increasing^1–5^. The electro-optical modulator is the key component in optical fiber communication, which modulates the light signal for loading information through electricity. Lithium niobate (LiNbO_3_, LN) exhibits a high-performance electro-optic effect and high optical transparency^6,7^. Therefore, LiNbO_3_ has been widely applied in electro-optic modulators. Generally, optical modulation is realized by a voltage-induced refractive index change, which can be described by the change in the ellipsoid of the refraction index influenced by an external electric field as $\Delta \beta _{ij} = \gamma _{ijk}E_k + h_{ijkl}E_kE_l + \ldots$, where Δβ_*ij*_ is the variation in the dielectric impermeability under the external electric field (*E*), γ_*ijk*_ is the linear electro-optic coefficient or Pockels coefficient, and *h*_*ijkl*_ is the quadratic electro-optic coefficient or Kerr coefficient. For LN, the linear electro-optic coefficient is *γ*_33_ = 30.9 × 10^−12^ mV^−1^, while other photonic materials usually have a very small or zero linear electro-optic coefficient. For example, the linear electro-optic coefficient of GaAs is *γ*_41_ = 1.5 × 10^−12^ mV^−1^, which is one-twentieth that of LN.

**Fig. 1:**
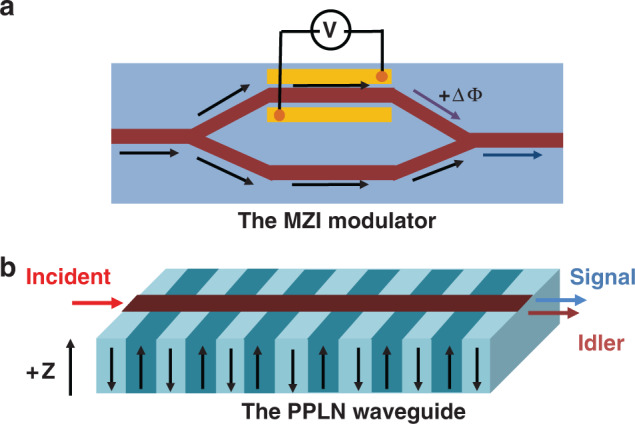
**a** Schematic of the Mach–Zehnder interferometer (MZI) modulator on LN. **b** Schematic of the periodically poled lithium niobate (PPLN) waveguide

An electro-optic modulator can be constructed on LN wafers by fabricating waveguides via defect engineering, including titanium diffusion or proton exchange, which is compatible with complementary metal–oxide–semiconductor (CMOS) technology. For example, the 50:50 Y-junction microstructure splits the input light into two LN optical waveguides that form the two arms of the Mach–Zehnder interferometer (MZI) modulator, a traditional electric-optical modulator. As shown in Fig. 1a, the applied voltage induces a phase delay in one arm and a phase advance in the other, which in turn changes the output intensity at the Y-combiner through interference^7^. The minimum voltage required to completely switch the output between on and off is defined as the half-wave voltage (*V*_π_). However, the low refractive index contrast of the titanium diffusion (Δ*n* ~0.01) or proton exchange (Δ*n* ~0.1) waveguides resulted in weak confinement and a large bending radius of the waveguides^8^. The weak optical confinement requires metal electrodes to be placed far from the optical waveguide, which in turn lowers the electro-optic efficiency. Hence, either large voltages or long electrodes are required to sufficiently modulate traditional LN modulators (with a size of approximately several centimeters). Therefore, the product of the half-wave voltage and electrode length, *V*_π_*L*, is a common figure of merit for electro-optic modulators. Nevertheless, the capacitance of the long electrodes limits the bandwidth, which results in a trade-off between the driving voltage and bandwidth^7^.

On the other hand, ferroelectric domain engineering of LN crystals has been extended from 1D to 2D and 3D, which was comprehensively reviewed in reference^9^. Periodically poled lithium niobate (PPLN) has been widely applied in the fields of frequency conversion, nonlinear beam shaping^10^, and quantum entanglement based on quasi-phase-matching (QPM) theory^11–15^. The second-order nonlinear optical coefficients (*d*_33_ and *d*_31_) of LN are 25.2 and 4.6 pm V^−1^ at 1064 nm, respectively^16^. Because LN is only poled along the *z*-axis, PPLN is appropriate for polarizing light to parallel to the *z*-axis to exploit the largest nonlinear-optical coefficient *d*_33_. As shown in Fig. 1b, a channel waveguide was fabricated on the surface of a PPLN wafer, which can confine the pump beam over the entire interaction length to greatly increase the conversion efficiency.

Moreover, considering the device size, reliability, cost, and energy consumption, photonic integrated circuit (PIC) technology has recently attracted significant interest in the rise of the integrated photonics field^17–23^. Integrated photonics is concerned with the integration of all the key components connected by a waveguide in a single chip (photonic platform) using a single material (monolithic integration) or multiple materials (hybrid integration). Most integrated photonic circuits have been built on four key platforms: indium phosphide^24,25^, silicon-on-insulator (SOI)^26–28^, silicon nitride^25,29^, and LN^30,31^. Nevertheless, no photonic platform has yet produced an optimal overall photonics system performance^17^. Generally, the selection of a material platform is based on the functionality of the optical components in the circuit, a low propagation loss, and industry-compatible fabrication processes^18^. For example, the compatibility of silicon integrated circuit manufacturing is the main reason for the development of silicon photonics^32^. Moreover, the manufacturing methods are suitable for the construction of SOI rib waveguides with a small cross-section of 1 μm^2^ with propagation losses as low as 0.1–0.5 dB cm^−1^ and a high index contrast between the Si waveguide layers (*n*_Si_ ~3.4) and SiO_2_ cladding layers (*n*_SiO2_ ~1.4), affording strong optical confinement and a small bending radius of 10 μm^33^. The low propagation loss and high index contrast of photonics systems, which make SOI one of the most active photonic platforms, are important evaluation standards for photonic platforms^34^. However, the 3 dB electrical modulation bandwidths of all-Si photonic devices have a theoretical limit of approximately 60 GHz^35^.

**Fig. 2:**
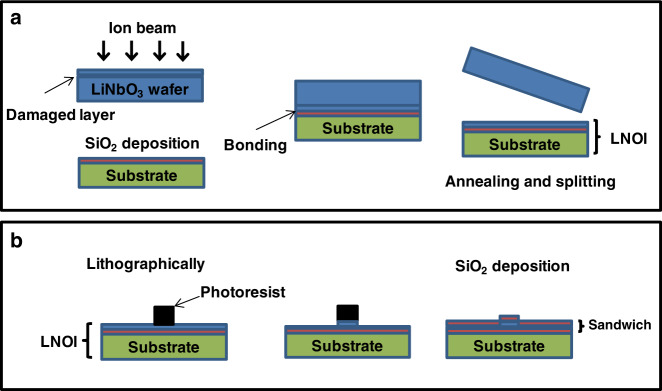
**a** Schematic of the preparation of LNOI. **b** Schematic of the microstructure engineering of LNOI

To conclude, LN is a promising photonic matrix due to its advantageous electro-optical effect and nonlinear optical properties. For example, on-chip generation and manipulation of entangled photons based on annealed proton exchanged waveguide circuits integrated on a z-cut PPLN crystal was demonstrated^36^. Unfortunately, bulk LN waveguides have large footprints, and the fabrication technology is not compatible with CMOS technology. Above all, nanophotonic LN waveguides with a larger refractive index contrast and a smaller optical mode size have emerged as a branch of integrated lithium niobate photonics^37–40^, although LN was perceived as a difficult-to-etch material. Over the past 25 years, integrated lithium niobate photonics have relied almost exclusively on high-quality lithium niobate thin film on insulator (LNOI) technology^41^ and advanced PIC technology for etching nanophotonic waveguides and microphotonic modulators. On the one hand, LNOI with a diameter of 3 in. can be fabricated by the smart cut technique^41^. In this technique, a submicron single-crystalline LN film is first detached from a sacrificial wafer by crystal ion slicing and then bonded with a SiO_2_-deposited LN substrate or Si substrate, forming LNOI (Fig. 2a). On the other hand, the CMOS-compatible microstructure engineering of LNOI for integrated photonic circuits, including nanophotonic waveguides and advanced functional modulators, has advanced considerably. Briefly, the microstructure of integrated photonic circuits is transferred to LN thin films by a lithographically defined mask. Then, the LN thin film is etched by Ar^+^ milling, forming thin slabs with a thickness of several hundred nanometers, which form ridge waveguides. Finally, a microscale-thick SiO_2_ cladding layer is deposited on top of the etched LN thin film by plasma-enhanced chemical vapor deposition (PECVD) to form a sandwich structure (Fig. 2b). The refractive index contrast between the LN core (*n*_LN_ ~2.1) and SiO_2_ cladding (*n*_SiO2_ ~1.4) is approximately 0.7, which is much larger than that of traditional LN waveguides; thus, LNOI can serve as strongly guiding planar waveguides even with a core layer of sub-micron thickness. Furthermore, domain engineering of LNOI was studied systematically for phase-matching nonlinear photonics. CMOS-compatible domain engineering of LNOI is gradually accelerating the development of integrated nonlinear photonics, which will play an important role in integrated photonic quantum technology^42^. In addition, considering the difficulty of etching LN, microstructure engineering using an easy-to-etch photonic material (e.g., SOI or silicon nitride) rib-loaded on LNOI is an attractive approach for hybrid integration of integrated photonic circuits, which not only obviates the necessity of etching LN but also combines the scalability of silicon photonics with the excellent electro-optic modulation of LN^43^.

Lithium niobate, as a traditional multifunctional material, has stimulated a photonics revolution as silicon did for electronics. Herein, we review the progress in microstructure and domain engineering of LNOI for integrated lithium niobate photonics, including photonic modulation and nonlinear photonics. Above all, ultra low-loss highly confined LN nanophotonic waveguides are the most fundamental building block^17^. Furthermore, the optical modulation device is at the heart of integrated lithium niobate photonic systems, as it encodes the RF signal. In Section “Microstructure engineering of LNOI for photonic modulation”, the progress in the microstructure engineering of LNOI for the preparation of ultra low-loss highly confined nanophotonic waveguides and microphotonic modulators is described. Furthermore, we review the progress in on-chip nonlinear photonics involving highly confined photonic microstructures and phase-matching techniques for LNOI in Section “Microstructure engineering of LNOI for nonlinear integrated photonics”. The focus is on the domain engineering of LNOI for QPM nonlinear photonics.

## Microstructure engineering of LNOI for photonic modulation

Generally, the basic microstructure of integrated photonic circuits for photonic modulation contains ridge waveguides and microphotonic structures, including micro rings, microdisks, and microracetracks. Whispering-gallery-mode (WGM) microresonators are the most compact micro modulator with high confinement of light into small ring volumes, such as microrings, microdisks, and microracetracks^44^. Optical WGM microresonators have some exceptional properties, such as a small mode volume, a high power density, and a high-quality factor *Q* (*Q* = *λ*/Δλ, where *λ* is the wavelength at which resonance occurs and Δ*λ* is the linewidth of the resonance wavelength). The refractive index changes when an electric field is applied to LN microresonators based on the electro-optic effect, which modifies the effective optical path length of the resonator. Thus, the resonance frequency of the microresonator shifts, thereby realizing electrical tuning.

In the early stage, efforts to construct microstructures on LNOI for photonic modulation were focused on low-loss nanophotonic waveguides and high-Q microresonators. Furthermore, MZI modulators integrated on LNOI platforms with advanced functionalities have been reported. In this section, both complementary microstructure engineering approaches for photonic circuits, i.e., direct etching of LNOI (monolithic integration) and using other photonic materials rib-loaded on LNOI (hybrid integration), are reviewed.

### Microstructure engineering by direct etching of LNOI

#### Z-cut LN films for TM polarization

**Fig. 3:**
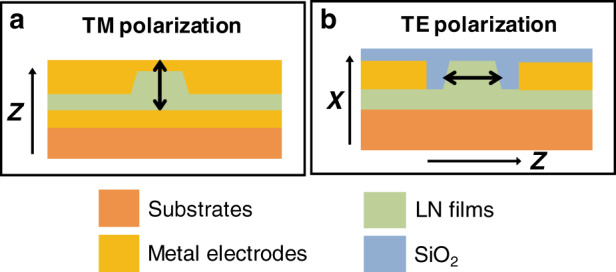
Schematic of both TM **a** and TE **b** polarization of LN nanophotonic waveguides on z-cut and x-cut LN films, respectively

Generally, the development of WGM microresonators for integrated lithium niobate photonics is accompanied by the improvement of the LNOI preparation technique. In the early stage, z-cut LN films were generally selected because of the limitations of lithographic techniques. Metal electrodes can be deposited between the substrate and the waveguide so that an electric field can be applied along the z-axis to exploit the largest electro-optic coefficient, *r*_33_, of LN, with light polarization along the *z*-axis (the electric field direction is mainly perpendicular to the LN film, which is called transverse magnetic (TM) polarization) (Fig. 3a).

**Fig. 4:**
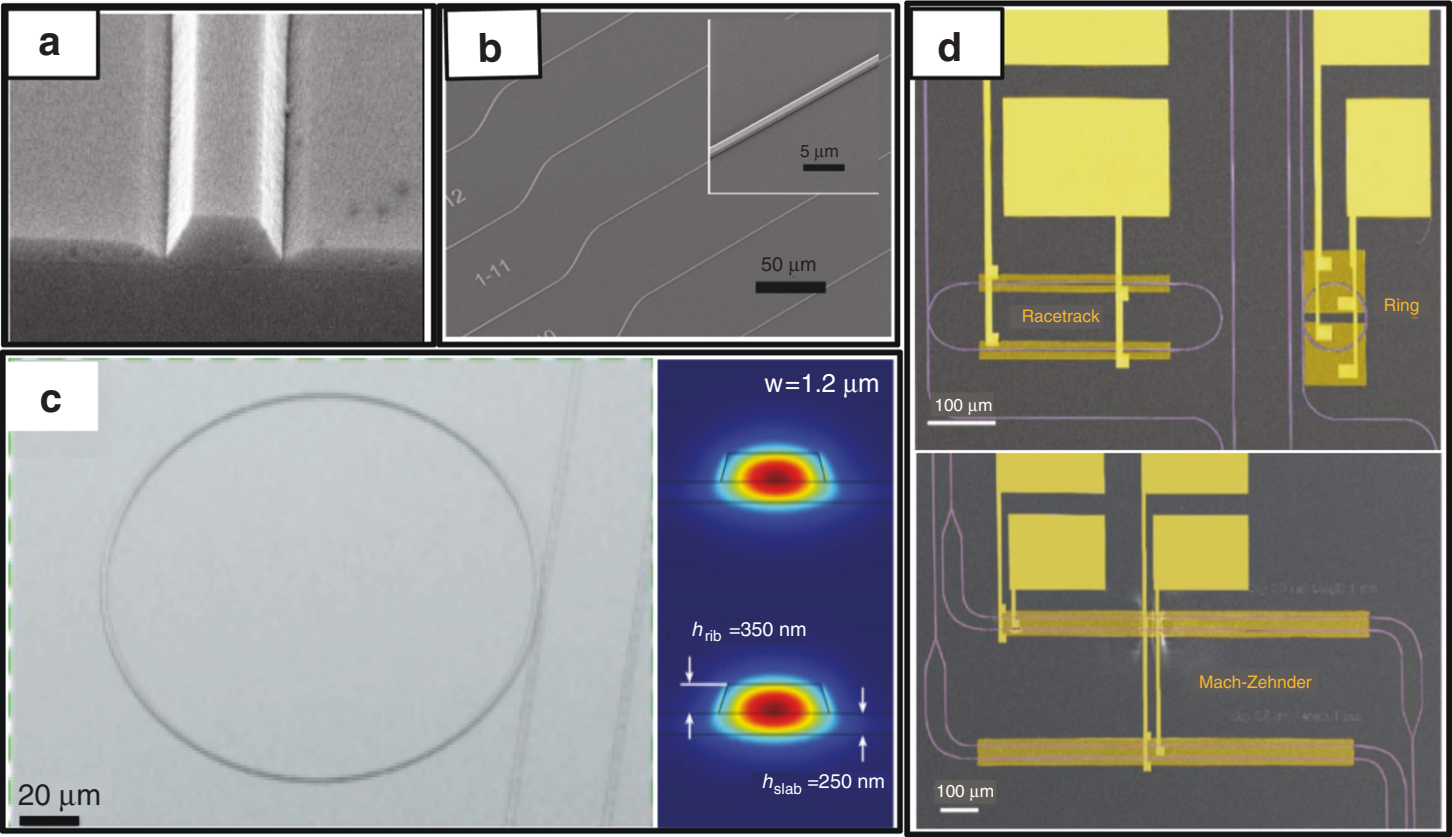
**a** Image of microchannel ridge guides (photonic wires) with a 1 μm top width^50,154^; Copyright 2009, Optical Society of America. **b** Array of LN waveguides with slightly different widths, with the inset showing a typical uniform LN waveguide with a fixed width^53^; Copyright 2017, Optical Society of America. **c** Image of a microring resonator consisting of dry-etched sub-wavelength waveguides with extracted propagation losses as low as 2.7 dB m^−1^ (left), as well as simulated optical modes in a bent (upper right) and straight (lower right) waveguides^55^; Copyright 2017, Optical Society of America. **d** Microphotonic devices including sub-micron waveguides, ring resonators, racetrack resonators, and MZI modulators integrated on an LNOI platform^54^; Copyright 2018, Optical Society of America

To the best of our knowledge, a slab waveguide on LNOI was prepared for the first time by crystal ion slicing and wafer bonding (called the smart cut method) in 2004^45^. The electro-optic coefficients and refractive index of the LN film were found to be comparable to those of the bulk LN crystal. Ridge waveguides on LNOI were first fabricated by photolithography and Ar^+^ beam milling^46^. However, the waveguide loss was not measured accurately and was estimated to be largely owing to the rough sidewalls caused by the etching process. It must be noted that the large mismatch in the thermal expansions of LN and SiO_2_/Si substrates can generate strong thermal stress during the bonding and layer transfer processes. Park et al.^47^ introduced laser irradiation for the layer transfer process to promote localized layer exfoliation on an LN donor substrate and obtained a 6 mm^2^ LNOI sample with a microdisk structure. Later, Guarino et al.^48^ introduced benzocyclobutene (BCB) as an adhesive layer between the metal electrode and LN film to reduce thermal stress and successfully prepared a centimeter-sized LNOI sample^49^. More importantly, an electro-optically tuneable WGM microresonator was introduced for the time on an LNOI platform. The transmission spectrum was tuned by the electro-optic effect, with a frequency tunability of 0.14 GHz V^−1^. However, BCB did not allow high-temperature annealing to repair the implantation-induced defects, which resulted in an electro-optical activity that was approximately 50% that of the bulk LN crystal and a large propagation loss of approximately 17 dB cm^−1^. Nevertheless, the study validated the viability of integrated lithium niobate photonics and stimulated further research on ultralow-loss sub-micron waveguides. Hu and co-workers prepared directly bonded LNOI without a BCB adhesive layer, which allowed high-temperature annealing to minimize ion-implantation-induced defects. Based on this, LN photonic wires with small cross-section dimensions of 1 × 0.73 μm^2^ were fabricated on an LNOI substrate (Fig. 4a) etched by inductively coupled plasma (ICP) Ar^+^ milling^50^. The measured propagation loss was 9.9 dB cm^−1^ at a 1.55 μm wavelength. Bo and co-workers fabricated LN microdisks with an undercut structure on an LNOI platform by UV photolithography, Ar^+^ plasma etching, and HF etching^51^. The microdisk resonator coupled with a tapered fiber exhibited a quality factor of 1.19 × 10^6^, which was two orders of magnitude higher than that of the reference microring resonator^48^.

#### X-cut LN films for TE polarization

However, z-cut LN films only work for TM polarization and are not suitable for on-chip integration. Therefore, x- or y-cut LN films were adopted, with light polarization along the *z*-axis (the electric field direction is mainly parallel to the LN film, which is called transverse electric (TE) polarization) to exploit the electro-optic coefficient *r*_33_. For this, metal electrodes must be deposited on both sidewalls of the waveguide channel, and an electric voltage should be applied along the z-axis of LN (Fig. 3b). As mentioned above, traditional LN modulators have weak optical confinement due to the low refractive index contrast. Therefore, metal electrodes are generally placed far from the waveguide to reduce the propagation loss due to metal absorption. Importantly, the much stronger optical confinement of channel waveguides in LNOI allows the electrodes to be placed close (micron-scale distance) to the sidewall of the waveguides, which results in a good overlap between the optical and microwave fields as well as strong phase modulation.

Bernal and co-workers improved the etching process to prepare a ridge waveguide with a smooth sidewall and reduced the propagation loss to 5 dB cm^−1^ on an x-cut LNOI platform^52^. Wang and co-workers used an etched amorphous silicon (a-Si) mask as a resist to transfer the pattern to x-cut LN layers followed by Ar^+^ milling. A uniform waveguide was prepared with a propagation loss of 3.0 ± 0.2 dB cm^−1^ ^53^. More excitedly, an array of LN waveguides with slightly different widths was successfully patterned on an LNOI platform with minimal surface roughness to obtain a manageable scattering loss, as shown in Fig. 4b. Later, microring resonators, racetrack resonators, and MZI modulators were integrated on the x-cut LNOI platform by directly shaping the LN thin films into sub-wavelength waveguide channels (Fig. 4d)^54^. For racetrack resonators, the electric fields applied to the two racetrack arms were in the same direction to double the phase shifts in the two arms. The electro-optic efficiency was measured as 7.0 pm V^−1^, which was similar to that of the ring resonator. For the MZI modulator, the half-wave voltage was 9 V with 2 mm-long electrodes. Thus, the product of the switching voltage and electrode length was 1.8 V cm, which is nearly an order of magnitude lower than that of traditional MZI modulators^7^. However, the propagation loss of the sub-wavelength waveguides of 3 dB cm^−1^ limits the application of the LNOI photonic platform, which arises because of the sidewall roughness caused by the etch mask.

By optimizing the ICP reactive-ion etching process for an LNOI platform, Zhang and co-workers fabricated sub-wavelength waveguides with a width of 1.2 μm and a low propagation loss of 2.7 dB m^−1^. Furthermore, they demonstrated a nearly critically coupled microring resonator with an ultrahigh Q of 5 × 10^6^ (Fig. 4b)^55^. This important finding expanded the applicability of the LNOI photonic platform. Using a similar etching process, an integrated MZI modulator with a half-wave voltage of 1.4 V was fabricated on an LNOI platform, which is compatible with CMOS drive voltages^56^. Furthermore, several MZI modulators with various microwave signal line widths and device lengths were successfully integrated on an LNOI platform. The integrated MZI modulator with a small size of several millimeters could overcome the voltage-bandwidth trade-off^57^, exhibiting very high bandwidths of up to 100 GHz. Moreover, an integrated MZI modulator on an LNOI platform demonstrated an unprecedented high electro-optic response of up to 500 GHz^58^.

In summary, the smart cut method for the preparation of wafer-scale LNOI samples (NanoLN, Jinan Jingzheng Electronics Co., Ltd.) greatly promoted the development of integrated lithium niobate photonics. On the one hand, the direct bonding between LN films and substrates without an adhesive layer allowed high-temperature annealing of the LNOI sample to minimize ion-implantation-induced defects. Then, the propagation loss of the nanophotonic LN waveguide was progressively reduced by optimizing the ICP reactive-ion etching process. On the other hand, the substitution of a z-cut LN film by an x-cut LN film greatly promoted the integration level of photonic circuits. The channel patterns with electrodes were easily transferred to an x-cut LN film, while the bottom electrode generally occupied the whole plane for a z-cut LN film. Importantly, the gold electrodes can be placed very close to the edge of a resonator on an x-cut LN film, which results in strong phase modulation without affecting the Q factor.

### Microstructure engineering using other photonic materials rib-loaded on LNOI

As mentioned above, no existing photonic platform can deliver an optimal overall MWP system performance. There are strict requirements for the laboratory equipment for the direct etching of LN. Thus, microstructure engineering for integrated photonic circuits is often carried out using other photonic materials rib-loaded on LNOI. Generally, this involves two-hybrid approaches: LN thin films are bonded onto silicon photonic circuits as a top cladding, and other photonic materials are rib-loaded on LNOI as a waveguide core. The electro-optic modulation under hybrid integration results from the overlap of the evanescent tail of the guided mode in the LN region.

#### LN films as top cladding

**Fig. 5:**
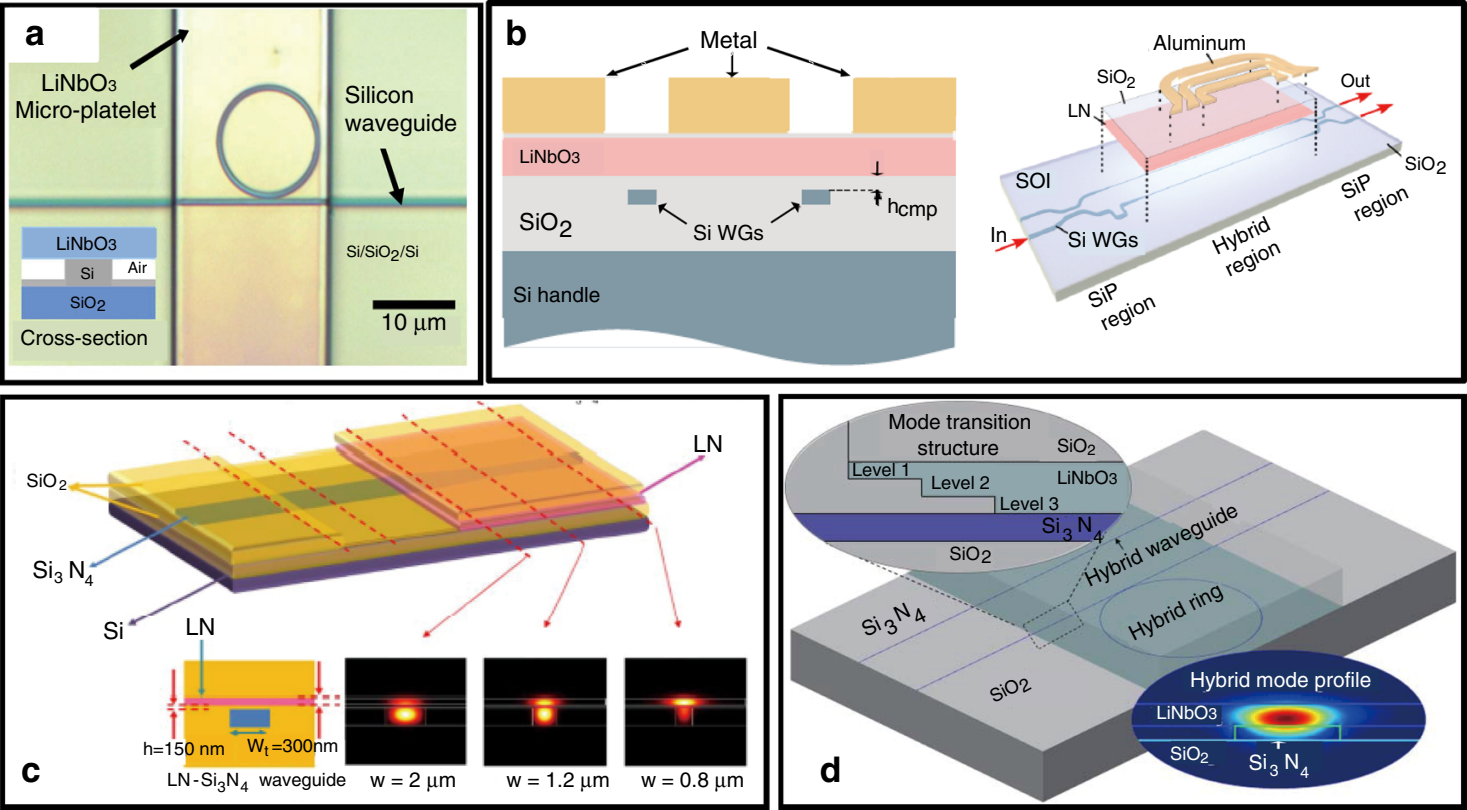
**a** Optical micrograph of a hybrid Si–LiNbO_3_ microring resonator^59^; Copyright 2011, Optical Society of America. **b** Schematic of the hybrid Si–LiNbO_3_ MZI modulator showing its cross-section^63^; Copyright 2018, Optical Society of America. **c** Schematic of the structure and cross-sections of hybrid Si_3_N_4_–LiNbO_3_ waveguides, and simulated profiles of the fundamental TE modes at 1540 nm^65^; Copyright 2017, Optical Society of America. **d** Schematic of hybrid Si_3_N_4_–LiNbO_3_ waveguides with a terrace shape^66^; Copyright 2018, Optical Society of America

In 2011, Lee et al.^59^ demonstrated hybrid Si-LiNbO_3_ electro-optically tunable ring resonators with free-standing z-cut LN thin films directly bonded to a Si microring resonator (Fig. 5a). The free-standing LN thin film was bonded onto the Si microring resonator as a top cladding, and an effective electro-optic coefficient of 1.7 pm V^−1^ was obtained for the TE mode, which is approximately one-fifth *γ*_13_ (8.6 pm V^−1^). Unfortunately, the largest electro-optic coefficient, *γ*_33_, was not exploited. Chen and Reano^60^ used BCB as an intermediate bonding layer to fabricate a hybrid Si–LiNbO_3_ microring resonator. Because of the absence of an integrated electrode, the voltage applied at both ends of the device was much larger, as the SiO_2_ layer consumed most of the voltage. Later, a hybrid Si–LiNbO_3_ microring resonator (z-cut LN thin film) with an integrated electrode was reported, which offered low-voltage tunability^61^. The microresonator showed a quality factor of 11,500 and exhibited a resonance tuning of 12.5 pm V^−1^ for the TM mode, which used the largest electro-optic coefficient of LN, *γ*_33_. Because of the limitation of the annealing temperature due to BCB, a pre-annealing process was employed to repair the crystal lattice and improve the electro-optic properties of the LN film. Here, the LN thin film was transferred onto a Si wafer and annealed at 1000 °C before bonding to the Si microring resonators. Thus, an electro-optic microresonator on a hybrid Si–LiNbO_3_ platform with a bandwidth of up to 5 GHz was demonstrated for the first time^62^.

However, the refractive index of Si (~3.4) is much higher than that of LN (~2.2), which dramatically decreases the confinement factor in the LN region. It was found by simulation that the fraction of the optical mode power in LiNbO_3_ is only 11% for the TE mode. Weigel et al.^63^ introduced a thin SiO_2_ layer (150 nm) between an x-cut LN thin film and a Si waveguide. As shown in the cross-section schematic of a hybrid MZI modulator (Fig. 5b), the LN thin film was oxide-bonded to the patterned and planarized silicon photonic circuits with a SiO_2_ interlayer. It was found that the thickness of the SiO_2_ layer has a significant impact on the bandwidth and *V*_π_*L* of MZI modulators. For this configuration, the fraction of the optical mode power in the LN region increased to 81%. The hybrid Si–LiNbO_3_ MZI modulator with a length of 0.5 cm achieved a 3 dB electrical modulation bandwidth of 106 GHz and a *V*_π_*L* of 6.7 V cm.

Compared with Si, Si_3_N_4_ has a slightly smaller refractive index (~1.98), a lower material loss, and a broader transparency window^64^. Chang et al.^65^ designed a hybrid Si_3_N_4_–LN waveguide by bonding LN thin films onto a Si_3_N_4_ waveguide layer on a Si substrate. As shown in Fig. 5c, it was found by simulation that the confinement factors of the two cores are sensitive to the Si_3_N_4_ ridge width, which is attributed to the approximate refractive index contrast between Si_3_N_4_ and LN. This configuration may be promising for a wide range of chip-level photonic applications.

However, the direct bonding of LN onto a predefined Si_3_N_4_ or Si waveguide resulted in a significant mode transition loss at the interface due to the substantial disparity in effective indices and mode profiles. Thus, a mode converter structure must be designed to achieve a low transition loss from the waveguide cores to the hybrid core-cladding waveguide region. As shown in Fig. 5d, an LN film with a terrace shape was etched and then bonded onto the Si_3_N_4_ waveguide on an SOI substrate. It was found by simulation that the transition loss was reduced from 2.67 to 0.81 dB at the interface^66^.

Furthermore, Cai and co-workers designed vertical adiabatic couplers (VACs), which transferred the optical powerfully, rather than partially, between the two layers^43^. For this configuration, a nanophotonic LN waveguide was designed on the top LN film, serving as a phase modulator, as shown in the cross-section schematic of the hybrid Si–LiNbO_3_ waveguide. The interfaces between the bottom silicon inverse tapers and top superimposed LN waveguides couple light up and down between the two layers, which are named VACs. After optimizing the parameters of the hybrid MZI modulator, it exhibited an insertion loss of 2.5 dB, a *V*_π_*L* of 2.2 V cm, an electro-optic bandwidth of at least 70 GHz, and modulation rates up to 112 Gbit s^−1^. Therefore, it had significantly increased modulation efficiency compared with the directly bonded waveguide.

#### LNOI as a substrate

**Fig. 6:**
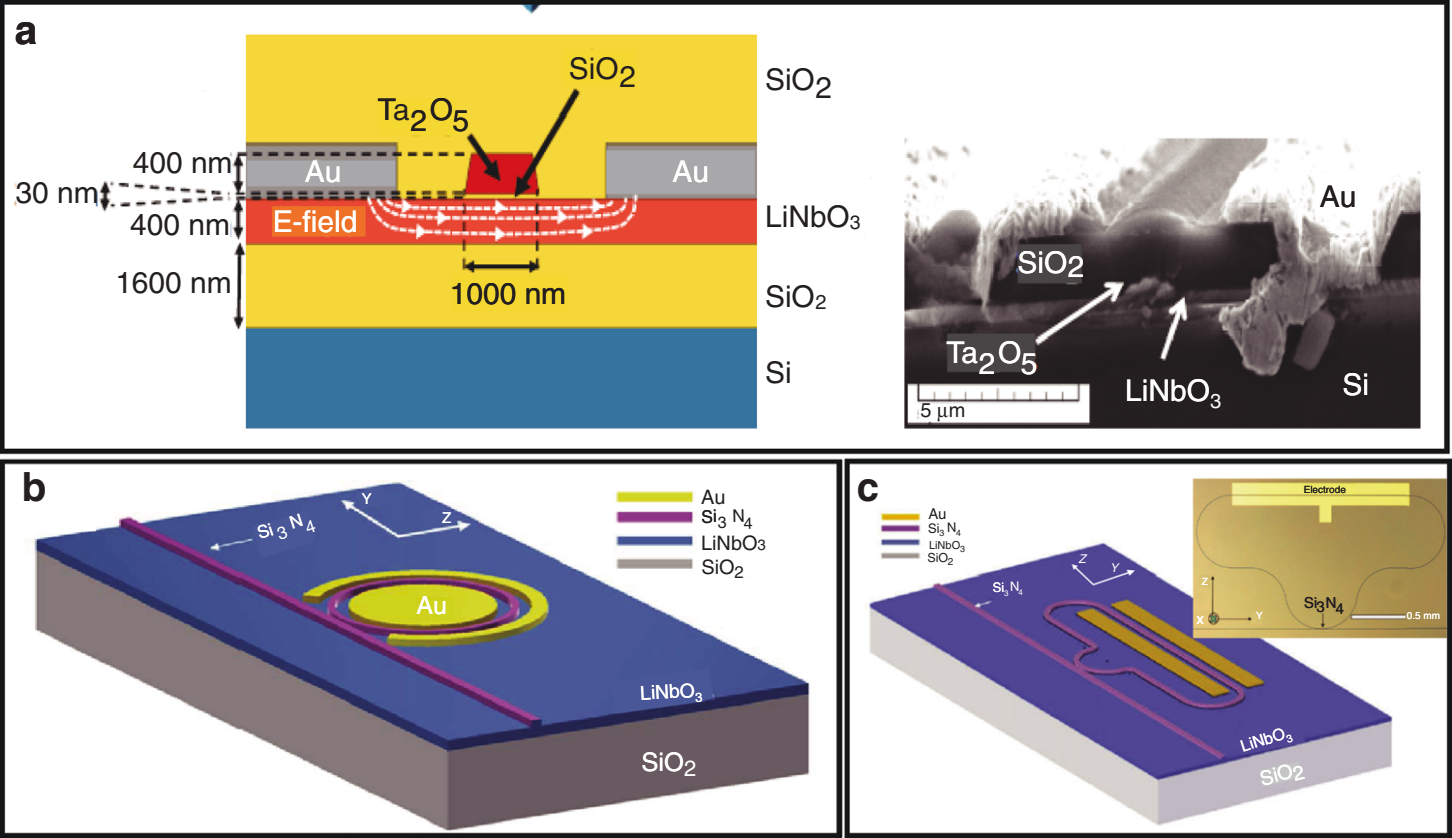
**a** Cross-section of the hybrid Ta_2_O_5_–LNOI waveguide^67^; Copyright 2013, Optical Society of America. **b** Schematic of the tuneable hybrid Si_3_N_4_–LNOI microring resonator^72^; Copyright 2019, Optical Society of America. **c** Schematic and microscope image of a tunable hybrid Si_3_N_4_–LNOI racetrack resonator^73^. Copyright 2019, Optical Society of America

Considering the difficulties in the direct etching of LN, LNOI can be used as a substrate with a rib-loaded material having a refractive index that forms a hybrid waveguide. Rabiei et al.^67^ designed a single-mode sub-micron ridge composite waveguide with a tantalum pentoxide (Ta_2_O_5_) rib region loaded onto an LNOI platform (Fig. 6a). The Ta_2_O_5_ rib region was prepared by selective oxidation of Ta^68^. Based on the composite waveguide, a microring resonator and an MZI modulator were prepared on a y-cut LNOI platform with a quality factor of approximately 7.2 × 10^4^ and a *V*_π_*L* of 4 V cm. Another index-matched material, Ge_23_Sb_7_S_70_ chalcogenide, was also used to fabricate a composite waveguide, which simplified the fabrication process and reduced the propagation loss to 1.2 dB cm^−1^ ^69^. Nevertheless, Ta_2_O_5_ and Ge_23_Sb_7_S_70_ chalcogenides are not conventional photonic materials and are incompatible with CMOS processing.

Hybrid Si–LN waveguides were fabricated by lithography on Si–LN platforms^70^, exhibiting a propagation loss of 2.5 dB cm^−1^ in the mid-infrared range. The *V*_π_*L* of the integrated MZI modulator was high at 27 V cm because of the large gap between the electrodes and the waveguide. In addition, silicon nitride was used to fabricate a composite waveguide by rib deposition on LNOI^71^. First, the silicon nitride layer was deposited on an LN film by PECVD. Then, ridge optical waveguides were patterned by standard lithography. Rao et al.^57^ prepared compact MZI modulators with a hybrid SiN–LNOI composite waveguide using a 2 μm-thick BCB layer as the top cladding. The high-performance devices showed a *V*_π_*L* of 3.1 V cm under DC and of less than 6.5 V cm with a bandwidth of up to 50 GHz. Furthermore, a tunable hybrid Si_3_N_4_–LN microring resonator (Fig. 6b)^72^ and a racetrack resonator (Fig. 6c)^73^ were lithographically deposited on an x-cut LNOI platform with a resonance tunability of up to 1.78 and 2.9 pm V^−1^, respectively. Both microresonators employed an air top cladding and exhibited a quality factor of 10^5^ for TE polarization.

In conclusion, the hybrid integration of photonic materials with an LN film is an effective approach for integrated photonics that can avoid the etching of LN. However, regardless of whether the LN film works as a top cladding or a substrate, the transition loss between the two layers is still a problem for this hybrid waveguide. Although the design of VACs nearly resolved the transition loss problem, the nanophotonic LN waveguide was still adopted, and the waveguide parameters must be precisely controlled for high modulation efficiency and low optical loss. This does not fully reflect the strengths of hybrid integration, which avoids the etching of LN. Therefore, the hybrid integration approach still needs further improvement.

## Microstructure engineering of LNOI for nonlinear integrated photonics

High-efficiency, compact, and integration-compatible wavelength converters using optical waveguides involve nonlinear integrated photonics^74–76^. In nonlinear optics, the polarization intensity of the dielectric is related to the intensity of the incident light wave as ${\mathrm{P}} = \varepsilon _0\left( {\chi ^{\left( 1 \right)}E + \chi ^{\left( 2 \right)}EE + \chi ^{\left( 3 \right)}EEE + \ldots\! } \right)$. The first term represents the first-order linear component, while the nonlinear optics is related to the higher-order components represented typically by the second- and third-order nonlinear optical susceptibilities (*χ*^(2)^ and *χ*^(3)^, respectively). The higher-order components are generally ignored. However, for nonlinear integrated photonics on semiconductor waveguides, third-order nonlinear optical interactions have been widely exploited because of some common photonic materials, such as SiO_2_ and Si, lack *χ*^(2)^, the second-order nonlinear optical susceptibility^75,77^. In addition, second-order nonlinear optical processes were investigated for some photonic materials, such as GaAs and AlGaAs, as *χ*^(2)^ is typically stronger than *χ*^(3)^ ^78^.

In the case of nonlinear integrated photonics on LNOI, second-order nonlinear frequency conversion has been widely researched because *χ*^(2)^ (linear electro-optic coefficient *r*_33_ = 3.09 pm V^−1^) is much larger than *χ*^(3)^ (the nonlinear refractive index is ~10^−15^ cm^2^ W^−1^ at 1064 nm) for LN^79^. Generally, photonic microstructures used for frequency conversion are designed based on strong mode confinement because the nonlinear effect can be significantly enhanced inside a small modal volume due to the increased field strength and temporal confinement of the interacting modes^80^. Thus, high-Q microresonators with light confined in a cavity for a long time serve as a promising avenue for nonlinear frequency conversion. Considering that the conversion efficiency of high-confinement resonators mainly depends on the modal overlap between the fundamental modes and higher harmonics^81,82^, strict phase matching is not required. However, obvious phase mismatch between the interacting waves often results from material dispersion. Therefore, several phase-matching techniques, as well as the associated waveguide microstructure engineering approaches, have been explored for second harmonic generation (SHG) processes, including modal phase matching (MPM), mode-shape modulation, and domain engineering^78^.

### Microstructure engineering of LNOI for phase-matching-free nonlinear optics

#### Microring cavities for frequency conversion

WGM microresonators as optical microcavities have been exploited for many different applications, including lasing on a chip, electro-modulation, nonlinear frequency conversion, and frequency comb generation^83–85^. Wang et al.^86^ demonstrated an on-chip integrated nonlinear frequency conversion process using microdisk resonators on an LNOI platform. The microdisk was undercut by wet etching using HF followed by electron beam lithography patterning and Ar^+^ milling. With the aid of a silica fiber to couple light into and out of the microresonators, on-chip SHG was obtained with a conversion efficiency of 0.109 W^−1^. In particular, spontaneous parametric down-conversion was achieved using microdisk resonators over a bandwidth of 400 nm^87^. A quasi-TM visible pump photon at a wavelength of 774.66 nm produced a pair of 1549.32 nm photons with orthogonal quasi-TM and quasi-TE polarizations, which is the operating principle of an entangled source. Nevertheless, microresonators with specific structures need to be designed to achieve highly efficient nonlinear frequency conversion^80^. Moreover, cascaded Raman scattering and frequency-doubled emission of Raman lines were observed at the microring resonators with a *Q* value of up to 10^6^ ^88,89^. An innovative tuneable coupling scheme to optimize the coupling was demonstrated for nonlinear optical processes by changing the distance between the coupling waveguide and the WGR in the z-direction.

#### Microring cavities have been used to realize optical frequency combs

Microring cavities have been used to realize optical frequency combs^90–92^, whose spectra consist of numerous discrete, equidistant laser lines, which was suggested to revolutionize wavelength division multiplexing in optical telecommunication. The currently available optical frequency combs are based on femtosecond lasers^93^. Nevertheless, further increasing the repetition rate into the frequency range above 10 GHz was an attractive challenge^94^. Recently, combs with wide spectra have been generated by the third-order Kerr nonlinearity (*χ*^(3)^) from a monolithic microresonator in some photonic materials^95–97^. However, the electro-optic modulator for tuneable filtering cannot be monolithically integrated into a single chip because of the lack of *χ*^(2)^. Wang et al.^98^ demonstrated frequency comb generation (*χ*^(3)^ microring resonator) and electro-optic manipulation (filtering and electro-optic modulation via a *χ*^(2)^ resonator) on a single integrated LNOI chip. The monolithic LNOI photonic circuits were patterned by directly shaping the LN thin films into a sub-wavelength waveguide channel by a similar standard process. A TE-polarized comb was generated from 1400 to 2100 nm with a line spacing of approximately 250 GHz. Meanwhile, different target comb lines can be selected by the *χ*^(2)^ microring resonator by applying different bias voltages. However, strong phase noise and stability are the most significant problems for Kerr frequency combs because of the complex nature of the third-order Kerr effect.

An electro-optic modulation is an alternative approach for producing optical frequency combs in a resonator with excellent stability and controllability^99^. This principle exploits the second-order nonlinearity involving a microwave field that modulates the optical wave within the nonlinear crystal. The resonance frequencies in the optical resonator are separated by the free spectral range (FSR), which must be designed to match the frequency of the microwave field, enabling sum- and difference-frequency generation of sidebands^100^. Rueda et al.^101^ demonstrated electro-optic comb generation based on an LN WGM resonator embedded in a copper microwave cavity. The WGM resonator was a convex-shaped disk with a radius of 2.45 mm and a thickness of 0.4 mm, resulting in an FSR of approximately 8.9 GHz at a pump wavelength of 1549 nm. With pump light of 320 μW and a low-power microwave modulation field of 20 dB at 8.9 GHz coupled into the WGM resonator, the generated comb spanned 11 nm with 180 lines in the C-band. It was demonstrated theoretically that strong phase modulation and high Q are crucial for microresonators to generate flat and broad electro-optic frequency combs^102^. Zhang et al.^102^ realized a broader electro-optic comb generator using high-Q microracetrack resonators integrated on a single LNOI photonic platform. The broad electro-optic frequency comb spanned 80 nm over part of the telecommunication C-band, the entire L-band, and part of the U-band with over 900 lines spaced by 10.453 GHz, which was generated by the resonator with a size of 200 μm × 6.2 mm modulated by a microwave field at a frequency near the resonator FSR of 10 GHz.

#### Metasurfaces

**Fig. 7:**
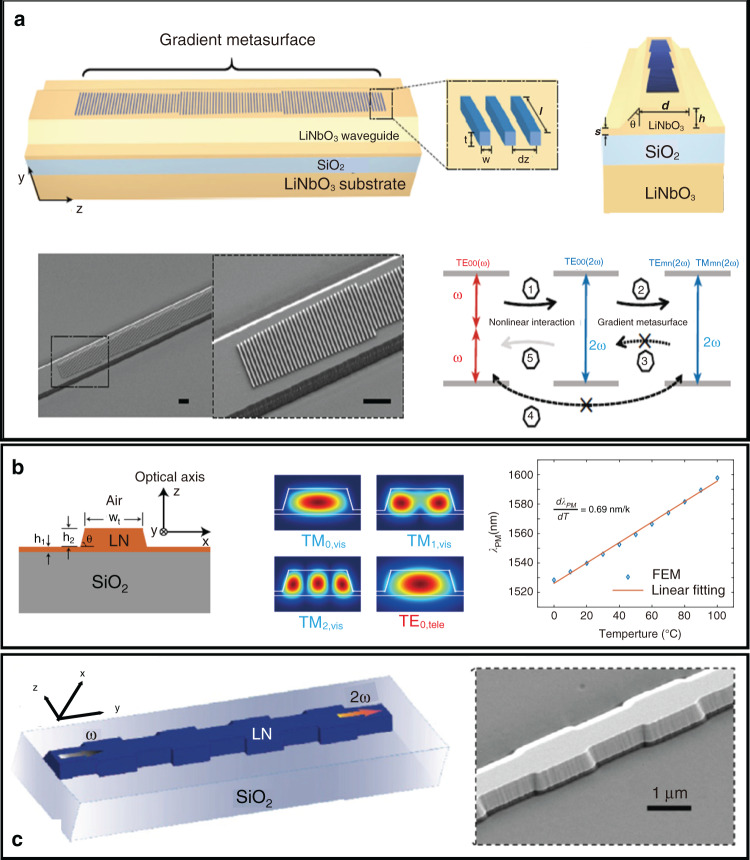
**a** Schematic and working principle of the metasurface of a LN nanophotonic waveguide^107^; Copyright 2017, Springer Nature. **b** Schematic and simulated mode profiles of a nanophotonic waveguide and the modal-phase-matching condition^111^; Copyright 2018, Optical Society of America. **c** Schematic and SEM image of the periodically grooved structure of a nanophotonic waveguide^53^; Copyright 2017, Optical Society of America

Metasurfaces consisting of nanoantennas are often used to realize enhanced optical nonlinearities^103–106^, as nonlinear plasmonic effects can arise from the coherent oscillations of conduction electrons near the surface of the metal structures^81^. A nanophotonic LN waveguide patterned with gradient metasurfaces was fabricated to achieve a monotonic increase in SHG power^107^. As shown in Fig. 7a, a gradient metasurface consisting of a number of identical phased antenna arrays was patterned along the top surface of the LN waveguide. Each array consisted of 35 amorphous silicon nanoantennas with a range of lengths. Generally, once optical power couples from the fundamental mode at the pump frequency, TE_00_(*ω*), to the fundamental mode at the SH frequency, TE_00_(2*ω*), it immediately starts to be converted into higher-order waveguide modes at the SH frequency, TE_mn_(2*ω*) and TM_mn_(2*ω*), by the gradient metasurface. Theoretically, the unidirectional wavevector provided by the gradient metasurface prevents optical power coupling from TE_mn_(2*ω*) and TM_mn_(2*ω*) modes back to the TE_00_(2*ω*) mode. Furthermore, the optical power carried by TE_mn_(2*ω*) and TM_mn_(2*ω*) modes cannot be coupled back to the TE_00_(*ω*) mode yet. Therefore, the gradient metasurfaces break the symmetry of the coupling between the pump and SH signals, resulting in high-efficiency SHG of approximately 1000% W^−1^ cm^−2^, which is three orders of magnitude more efficient than the bare LN waveguide.

### Microstructure engineering of LNOI for phase-matching nonlinear optics

In the most generic second-order nonlinear optical process, which is taken as an example here, three-wave mixing must satisfy energy conservation and momentum conservation.

$$\omega _3 = \omega _1 + \omega _2\,{\mathrm{and}}\,k_3 = k_1 + k_2$$

where $$k_i = n_i\omega _i/c$$

Thus, momentum conservation becomes

$$n_3\omega _3 = n_1\omega _1 + n_2\omega _2$$

The three waves travel at different velocities due to the dispersion of the medium, corresponding to the different refractive indices *n*_1_, *n*_2_, and *n*_3_. As a result, the refractive indices for the three waves need to be precisely controlled for phase matching.

#### Modal phase matching

MPM is a simple technique for phase velocity synchronism in nonlinear frequency conversion processes^108^. Because longer wavelengths have lower effective indices and the higher-order mode of the wavelengths has a lower effective index in a multimode waveguide, the fundamental modes of one or two of the longer wavelengths are phase-matched with a higher-order mode of shorter wavelength^78^. Generally, the type-0 configuration is employed to achieve a high conversion efficiency. Wang and co-workers demonstrated the dependence of the effective mode indices (*n*_eff_) of both the fundamental mode at the incident wavelength and higher-order modes at the SH wavelength on the top width (*w*_t_) of the LN waveguide with fixed initial thickness and sidewall angle. To exploit the large nonlinear coefficient *d*_33_, phase matching was achieved between the first-order TE modes at the fundamental wavelength and the third-order TE modes at the SH wavelength, with normalized conversion efficiencies as high as 41% W^−1^ cm^−2^ at *w*_t_ = 630 nm^53^. In addition, MPM was demonstrated between the fundamental TM mode at 1550 nm and the second-order TM modes at the SH wavelength on monolithic z-cut LNOI by changing the top width of the LN waveguide to approximately 590 nm^109^. SHG of the type-I configuration was also found for a nanophotonic LN waveguide between the fundamental quasi-transverse-electric mode (TE_00_) and the higher-order quasi-transverse-magnetic SH mode (TM_mn_). This waveguide inevitably exhibited a low conversion efficiency due to the significantly weaker nonlinearity (*d*_31_) than in the type-0 configuration (*d*_33_). On a sub-micron LN waveguide with a cross-section of 1.2 μW × 0.53 μW, the phase-matched SHG process occurs between the fundamental mode (TE_00_) and the second-order SH mode (TM_20_) at a wavelength of 1413 nm^110^. This SHG process generates an SH power of approximately 305 pW with an incident power of 737 μW, achieving a normalized SHG efficiency of approximately 6.9% W^−1^ cm^−2^.

Even so, the type-I configuration was often employed for wavelength tunability of SHG because LN exhibits a significant thermo-optic birefringence with a value of approximately 4 × 10^−5^ K^−1^ at room temperature. Thus, a temperature change of the device would result in a considerable variation in the material birefringence, which in turn shifts the phase-matched wavelength of the type-I SHG process. Based on the thermo-optic birefringence of LN, highly tuneable efficient SHG with a tuning slope of 0.84 nm K^−1^ was demonstrated in a z-cut LN nanophotonic waveguide by phase-matching the fundamental quasi-TE mode in the telecom band with the third-order quasi-TM mode at the SH wavelength (Fig. 7b)^111^. The waveguide exhibited a theoretical normalized conversion efficiency of 22.2% W^−1^ cm^−2^ and an experimentally determined SHG efficiency of 4.7% W^−1^ for type-I intermodal phase matching.

#### Mode-shape modulation

is an alternative poling-free implementation for integrated QPM with periodic gratings^112^. Waveguides with periodically grooved structures were also fabricated on an x-cut LNOI platform, which was adopted to realize phase-matched SHG (Fig. 7c)^53^. The periodic perturbation in the periodically grooved waveguide generates space harmonics with new propagation constants to compensate for the phase mismatch^113^. Moreover, a hybrid SiN_*x*_–LNOI waveguide in which the width of the SiN rib was periodically modulated was fabricated to achieve QPM SHG^114^.

### Domain engineering of LNOI for phase-matching nonlinear optics

In QPM theory, the phase variation is $\Delta k_Q = k_3 - k_2 - k_1 - k_{{\Lambda }}$, where the grating vector is $k_{{\Lambda }} = 2\pi /{{\Lambda }}$ for grating period ${{\Lambda }}$analogous to wave vectors. The nonlinear coefficient is modulated with a period twice the coherence length (*l*_*c*_) of the interaction to offset the accumulated phase mismatch. The grating period is determined from the relevant radiation frequency and the working temperature according to the equation ${{\Lambda }} = 2{\mathrm{l}}_c = \frac{{2\pi }}{{k_3 - k_2 - k_1}}$. Owing to the advantage of LNOI in integrated photonics, domain engineering of LNOI has attracted much interest for integrated frequency-conversion devices. The recently developed integrated frequency-conversion devices on PPLN films also involve hybrid and monoclinic approaches. For the hybrid approach, another photonic material was integrated on the PPLN film to guide the light, while frequency conversion was realized in the PPLN region. For the monoclinic approach, the ridge waveguide was etched in the PPLN region to form nanophotonic PPLN waveguides.

#### PPLN waveguides on LNOI platforms

**Fig. 8:**
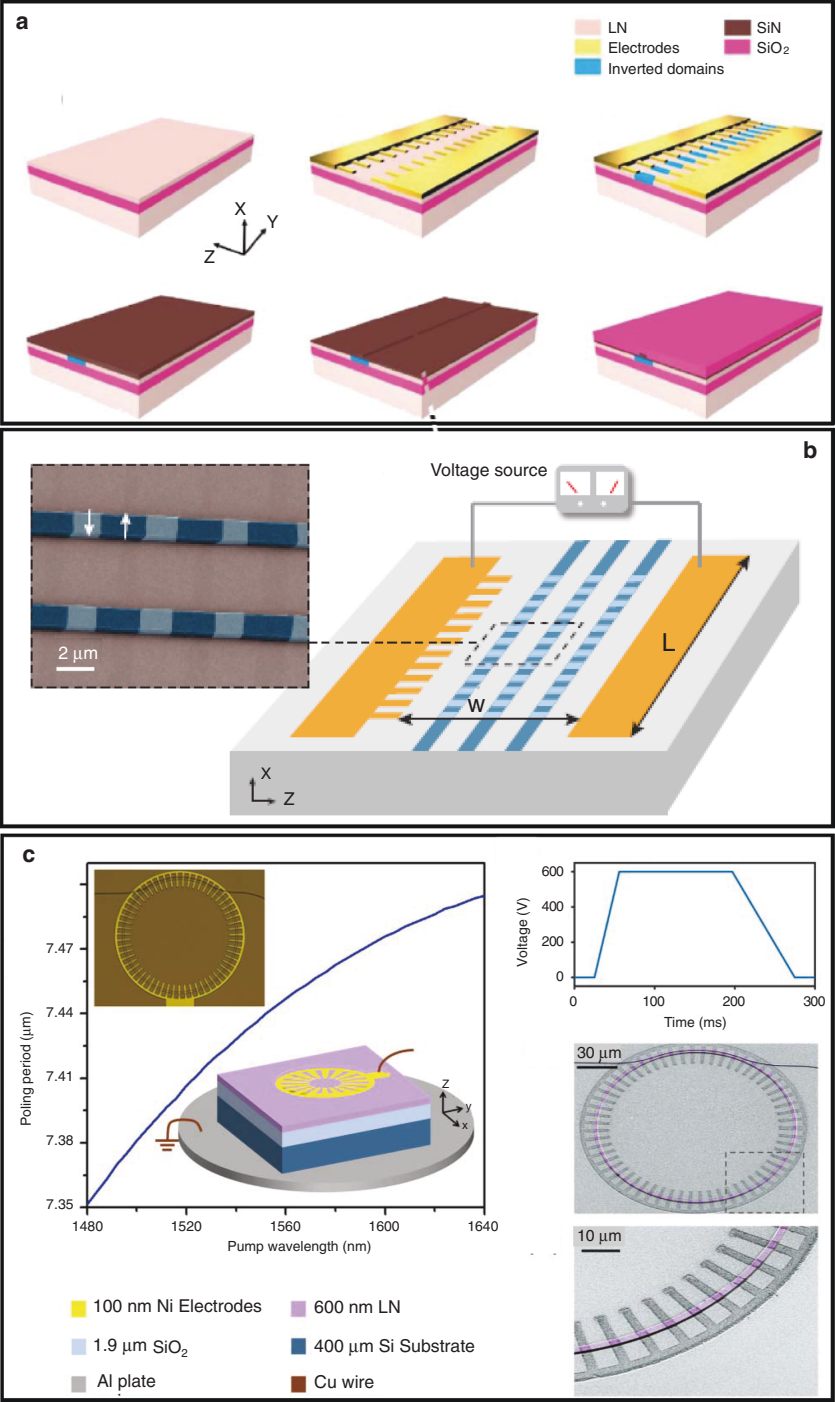
**a** Schematic of the processing of a hybrid SiN_*x*_–PPLN waveguide^118^; Copyright 2016, Optical Society of America. **b** Schematic and false-color SEM image of a periodically poled nanophotonic waveguide^120^; Copyright 2018, Optical Society of America. **c** Periodically poled LN microring resonator^128^; Copyright 2019, Optical Society of America

PPLN waveguides on LNOI platforms are crucial for realizing highly efficient, compact, and integration-compatible wavelength converters. Generally, PPLN is prepared by applying an external electric field using periodic electrodes. Gainutdinov and co-workers realized periodic poling of z-cut LN thin films on insulators using an atomic force microscope^115^, which is favorable only for the TM polarization mode to exploit the large nonlinear coefficient *d*_33_. Hence, the x- or y-cut geometry is preferred because of the possibility of ease of processing on top of the thin film sample. Mackwitz et al.^116^ first fabricated periodically poled domain patterns in x-cut LNOI. Rao et al.^117^ deposited poling electrodes on the same surface containing LN by filling the spaces of the pre-etched LN thin film, which can reduce the consumption of the electric field component parallel to the *x*-axis. In the early stages, the approach of hybrid integration was adopted for photonic materials rib-deposited onto PPLN films. Hybrid SiN_*x*_–PPLN waveguides were formed by rib-loaded channels of SiN_*x*_ in the periodic domain region of LN thin films, with a final deposition of a SiO_2_ top cladding, as shown in Fig. 8a. It was found by simulation that the SiN_*x*_ ribs with a cross-section of 2000 nm × 400 nm resulted in a poling period of approximately 5 μm for TE-polarized pump light at 1580 nm phase matched with the SH wavelength. Chang et al.^118^ also achieved QPM SHG with an output power of 80 nW at a 1530 nm pump power of 0.5 mW using hybrid SiN_*x*_–PPLN waveguides. The SHG exhibited a peak-normalized efficiency of 160% W^−1^ cm^−2^. The duty ratio of the periodically poled domain patterns in the x-cut LN thin film still needs to be improved^119^. Although the hybrid waveguide obviated the necessity of LN etching, it was difficult to enhance the nonlinear conversion efficiency. Monolithic nanophotonic PPLN waveguides propagating along the *y*-axis were successfully prepared by standard lithography on a periodically poled x-cut Mg-doped LN film (Fig. 8b)^120^. The nanophotonic PPLN waveguides with a cross-section of 1400 nm × 600 nm exhibited a domain-inverted period of ~4 nm, which indicated that the fundamental TE modes at 1550 nm were phase matched with the fundamental TE modes at the SH wavelength. Moreover, SHG of 117 mW at 775 nm was achieved using a pump power of 220 mW with an absolute conversion efficiency of 53% in a 4 mm-long device, corresponding to the normalized conversion efficiency of 2600% W^−1^ cm^−2^. With an optically monitored iterative poling, depoling, and repoling sequence to improve the domain-inverted structure of nanophotonic LN waveguides, conversion efficiency of up to 4600% W^−1^ cm^−2^ was achieved for SHG by pumping at approximately 1540 nm^121^.

#### Periodically poled microresonators

In addition, a PPLN thin film was exfoliated from a bulky PPLN wafer by the crystal ion slicing method^122,123^. It may have potential applications in nonlinear integrated photonics, such as micro rings and microdisks with periodic domain structures demonstrating QPM SHG^124–127^. A disk was fabricated by diamond polishing from a periodically poled LN wafer, which exhibited a maximum output power of 12.3 mW at a wavelength of 775 nm, as observed by a frequency-doubling experiment. The pump power at the input of the cavity with a wavelength of 1.55 μm was 25 mW, with a maximum SHG conversion efficiency of approximately 0.5^127^. In addition, the z-cut LN microring can be periodically polled by an external electric field between the bottom aluminum plate electrode and the top radial nickel electrodes (Fig. 8c)^128^. The results for the periodically poled microring with a period of 7.46 μm indicated that the fundamental TE mode was quasi-phase-matched with the SH TM mode to exploit the nonlinear coefficient *d*_31_. By coupling with a pulley bus waveguide, the periodically poled microring resonator was pumped at approximately 1617 nm, which yielded a QPM SHG conversion efficiency of up to 250,000% W^−1^ and absolute conversion efficiency of 15%. The higher SHG efficiency is attributed to the stronger mode confinement of the microring due to the larger modal overlap as well as to the phase-matching due to the periodic domain structure.

In conclusion, LNOI is a promising platform for nonlinear integrated photonics. Generally, the phase-matching condition is a critical factor in nonlinear frequency conversion processes. However, phase-matching-free SHG has been achieved with high-efficiency conversion by a gradient metasurface on an on-chip integrated nonlinear photonic device. This finding will promote the development of nonlinear integrated photonics on LNOI. Of course, many integrated nonlinear photonic devices were fabricated by following phase-matching SHG, especially quasi-phase-matching. The preparation of PPLN films greatly promoted the nonlinear integrated photonics on LNOI, especially the nanophotonic PPLN waveguides, which realized a conversion efficiency of up to 4600% W^−1^ cm^−2^. In the future, nanophotonic PPLN waveguides will be applied in quantum technology to develop integrated quantum technology.

## Summary and prospects

**Fig. 9:**
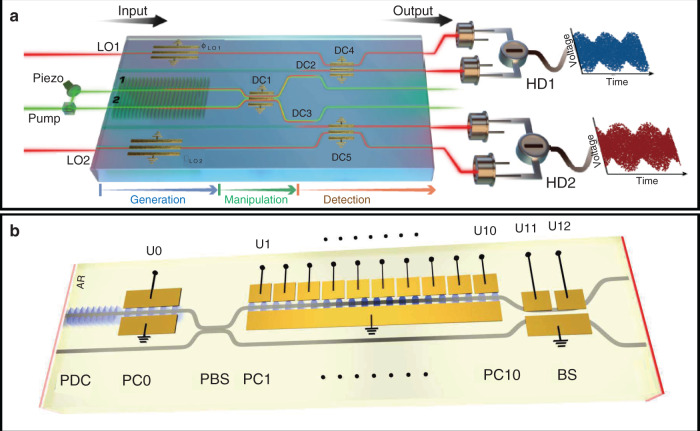
**a** Schematic of integration with the generation, manipulation, and interferometric stages of homodyne detection of nonclassical light on a H:LiNbO_3_ waveguide chip^129^; Copyright 2018, American Association for the Advancement of Science. **b** Schematic of integration with the photon-pair generation, propagation, electro-optical path routing, and voltage-controllable time delay on a single Ti:LiNbO_3_ waveguide chip^130^; Copyright 2019, American Association for the Advancement of Science

The recent progress in the microstructure and domain engineering of LNOI for integrated photonics was reviewed. It is feasible that microstructures can be constructed on LNOI platforms for photonic circuits, which are also compatible with CMOS technology. Ultralow-loss nanophotonic LN waveguides and high-Q WGM microresonators have been prepared on the LNOI platform, which enables the sophisticated manipulation of light signals. Compared with other photonic materials, LiNbO_3_ exhibits a high-performance electro-optic effect and nonlinear optical properties. On the one hand, the high-bandwidth and low-drive-voltage electro-optical modulator is a critical component in optical fiber communication, which can achieve high-speed signal conversion between electricity and light waves with low power consumption. The integrated modulator on LNOI has been realized experimentally with a 100 GHz bandwidth at a drive voltage of approximately 1 V, while the conventional LN modulators consume high power with a drive voltage of 3–5 V. This exciting progress indicates that LN modulators are likely to be manufactured in the mid to long term. On the other hand, PPLN waveguides have been widely applied in nonlinear optics, while domain engineering of LNOI has emerged as a new branch of nonlinear integrated photonics. Nonlinear photonics plays a very important role in quantum technologies, which comprise an emerging class of devices capable of controlling the superposition and entanglement of quantum states of light, to realize fundamental performance advantages over ordinary classical devices. The entanglement photon-pair source and single-photon detector are very important components of quantum technologies, which can be achieved through nonlinear integrated photonics on LNOI. Considering the low-loss propagation waveguide and high-bandwidth integrated modulators achieved on LNOI, LNOI will be a promising matrix for integrated quantum technologies, with the entanglement photon-pair source and photon detector integrated on an optical chip (Fig. 9a, b)^129–133^.

Thus, we predict that integrated quantum photonics will enable the generation, processing, and detection of quantum states of light at increasing scale and level of complexity. Integrated quantum photonics can provide programmable devices approaching 1000 components occupying only millimeter-scale footprints with the integrated generation of multiphoton states. For instance, quantum computers are suggested to be the most remarkable and powerful future quantum technology^134^. The nonlinear integrated photonics approaches for single-photon sources and detectors in optical quantum computers will be determined by their efficacy and practicality^131^. Quantum communication aims to realize quantum key distribution (QKD) transmitters and receivers based on the laws of quantum mechanics for security, which is inaccessible by traditional communications^135,136^. On-chip integration with necessary components, such as tunable lasers, attenuators, and electro-optic phase modulators, can lead to the realization of QKD transmitters, while on-chip integration with single-photon detectors and phase shifters can lead to the realization of QKD receivers.

Last, topological photonics may become an important application branch in integrated lithium niobate photonics. The topology, an indispensable degree of freedom of photonic systems that characterize the quantized global behavior of wavefunctions, can be optimized^137,138^. Topological effects can be realized in photonic crystals, coupled resonators, metamaterials and quasicrystals. Silicon photonics has been widely used to study topological features, whereas arrays of coupled optical resonators provide a toolbox to observe the wavefunction^139,140^. A “photonic molecule” with two distinct energy levels has been demonstrated using coupled microring resonators on LNOI, which can be controlled by external microwave excitation^141^. We predict that dynamically controlled multilevel photonic systems will promote research on topological photonics. Therefore, topological photonics on LNOI will open a new avenue for designing and controlling the behavior of light and possibly provide revolutionary applications.

The high quality and homogeneity of LN films are undoubtedly the most fundamental requirements for any photonic application. At present, the smart cut method is the main approach to prepare a submicron-thickness LN film. This method cannot meet the demands of large-scale manufacturing yet owing to the ion-implantation-induced defects and production efficiency. Therefore, the preparation approach of LNOI still needs to be improved and explored. The machine thinning method was also reported for the preparation of LN films^142^. The LN wafer can be polished to micron-scale and even submicron-scale films after bonding to Si substrates. It was reported that a micron-scale LN film was fabricated by this machine thinning method. An LN ridge waveguide with a cross-section of 1.3 μm × 4 μm was fabricated on this film^143^. With an electric field applied along the z-axis, electro-optic modulation was demonstrated with a *V*_π_*L* of 7.1 V cm, which is less than half that of a bulk LN modulator^142^. An LN modulator integrated onto a Si substrate using a CMOS-compatible process exhibited a frequency response of up to 110 GHz^144^. First, the thinner the machined LN wafer is, the higher the confinement achieved by the LN ridge waveguides. Second, the machine thinning method does not involve ion-implantation-induced defects. Thus, the machine thinning method should be improved, and submicron LN films will be fabricated through this method. Thus, the machine thinning method may be an alternative approach for fabricating high-quality LN films.

Until now, LN films have generally been exfoliated from nondoped congruent LN crystals. The congruent LN crystals have a large coercive field (21 kV mm^−1^) and a low optical damage threshold because of intrinsic defects^145^. An electric field of up to 40 kV mm^−1^ is needed to invert the ferroelectric domain in an x-cut LN film for QPM nonlinear integrated photonics. Therefore, an Mg-doped LN film was adopted in integrated photonics, which reduced the poling electric field to 7.6 kV mm^−1^. The waveguide showed no photorefractive damage at a high SH optical intensity of ∼10 MW cm^−2^ after many hours of optical pumping^120^. Furthermore, stoichiometric LN crystals have a smaller coercive field of ~3.5 kV mm^−1^ and a completely crystalline structure^146–152^. Therefore, stoichiometric LN films should be more suitable for ultralow-loss nanophotonic waveguides^153^ and can be poled with a smaller electric field.

Over the last decade, integrated lithium niobate photonics has been rapidly developed and proven invaluable in the development of future optical communication and quantum technologies. The large-scale and low-cost manufacturing of integrated photonic devices and systems by mature manufacturing processes will enable new revolutionary applications in optical communication and quantum technologies.
